# Programmed ventricular stimulation for risk stratification in symptomatic athletes with nonischemic left ventricular scar and preserved ejection fraction

**DOI:** 10.1093/europace/euag160

**Published:** 2026-09-08

**Authors:** Luigi Sciarra, Fabrizio Ricci, Elena Cavarretta, Zefferino Palamà, Alessio Borrelli, Mauro Di Roma, Leonardo Pignalosa, Federico Zanin, Daniele Falco, Giulia Renda, Cesare Mantini, Sabina Gallina, Francesco Brancati, Silvio Romano, Antonio Scarà

**Affiliations:** Department of Life, Health and Environmental Sciences (MeSVA), University of L'Aquila, Piazzale Salvatore Tommasi 1, 67100 L'Aquila, Italy; Department of Neuroscience, Imaging and Clinical Sciences, G. D’Annunzio University of Chieti-Pescara, Chieti 66100, Italy; Department of Medical-Surgical Sciences and Biotechnologies, Sapienza University of Rome, Latina 04100, Italy; Department of Cardiology, Casa di Cura Villa Verde, Taranto 74121, Italy; Department of Life, Health and Environmental Sciences (MeSVA), University of L'Aquila, Piazzale Salvatore Tommasi 1, 67100 L'Aquila, Italy; Cardiology Unit, GVM Care and Research, San Carlo di Nancy Hospital, Rome 00100, Italy; Department of Radiology, Aurelia Hospital, Rome 00100, Italy; Department of Life, Health and Environmental Sciences (MeSVA), University of L'Aquila, Piazzale Salvatore Tommasi 1, 67100 L'Aquila, Italy; Cardiology Unit, GVM Care and Research, San Carlo di Nancy Hospital, Rome 00100, Italy; Cardiology Unit, GVM Care and Research, San Carlo di Nancy Hospital, Rome 00100, Italy; Department of Neuroscience, Imaging and Clinical Sciences, G. D’Annunzio University of Chieti-Pescara, Chieti 66100, Italy; Department of Neuroscience, Imaging and Clinical Sciences, G. D’Annunzio University of Chieti-Pescara, Chieti 66100, Italy; Department of Neuroscience, Imaging and Clinical Sciences, G. D’Annunzio University of Chieti-Pescara, Chieti 66100, Italy; Department of Neuroscience, Imaging and Clinical Sciences, G. D’Annunzio University of Chieti-Pescara, Chieti 66100, Italy; Department of Life, Health and Environmental Sciences (MeSVA), University of L'Aquila, Piazzale Salvatore Tommasi 1, 67100 L'Aquila, Italy; Human Functional Genetics Laboratory, IRCCS San Raffaele Roma, Rome, Italy; Department of Life, Health and Environmental Sciences (MeSVA), University of L'Aquila, Piazzale Salvatore Tommasi 1, 67100 L'Aquila, Italy; Department of Life, Health and Environmental Sciences (MeSVA), University of L'Aquila, Piazzale Salvatore Tommasi 1, 67100 L'Aquila, Italy; Cardiology Unit, GVM Care and Research, San Carlo di Nancy Hospital, Rome 00100, Italy

**Keywords:** Programmed ventricular stimulation, Electrophysiological study, Nonischemic myocardial scar, Ventricular arrhythmias, Risk stratification, Preserved ejection fraction

## Abstract

**Aims:**

Non-ischaemic left ventricular (LV) scar is increasingly recognized in athletes with preserved left ventricular ejection fraction (LVEF) and may represent a substrate for malignant ventricular arrhythmias. The role of electrophysiological study (EPS) with programmed ventricular stimulation (PVS) for risk stratification in this population remains unclear. This study aimed to evaluate the prognostic value of inducibility at EPS in symptomatic athletes with non-ischaemic LV scar and preserved LVEF.

**Methods and results:**

We prospectively enrolled 72 consecutive athletes (mean age 48.9 ± 13.1 years; 66.7% male) with documented non-ischaemic LV scar, preserved LVEF (≥50%), and arrhythmic symptoms (tachycardic palpitations, syncope/presyncope, and/or exercise-related ventricular arrhythmias) undergoing EPS. Inducibility was defined as sustained ventricular arrhythmias during PVS. The primary endpoint was the occurrence of major arrhythmic events (MAE), including sudden cardiac death, sustained ventricular tachycardia/ventricular fibrillation, or appropriate implantable cardioverter-defibrillator therapy. Twenty-three athletes (31.9%) were inducible (EPS+), while 49 (68.1%) were non-inducible (EPS−). Baseline characteristics were largely comparable between groups, although QRS duration was longer in inducible athletes (104.6 ± 15.2 ms vs. 95.5 ± 14.3 ms; *P* = 0.019). Twenty-three inducible athletes underwent ICD implantation (18 transvenous and 5 subcutaneous devices), whereas 16 non-inducible athletes received an implantable loop recorder. During a median follow-up of 42 months, 11 major arrhythmic events occurred. Nine events occurred among inducible athletes and consisted of sustained ventricular arrhythmias (monomorphic VT, polymorphic VT, or ventricular fibrillation) successfully terminated by appropriate ICD therapy, whereas two major arrhythmic events occurred among non-inducible athletes and required successful resuscitation followed by ICD implantation. No arrhythmic deaths occurred during follow-up. Event-free survival at 50 months was 40.6% in inducible athletes and 95.9% in non-inducible athletes (log-rank *P* < 0.001).

**Conclusion:**

In symptomatic athletes with non-ischaemic LV scar and preserved LVEF, inducibility at EPS identifies a subgroup at higher risk of MAE, whereas non-inducibility is associated with a low event rate. EPS may represent a useful adjunctive tool for arrhythmic risk stratification in athletes, particularly in those without conventional high-risk features.

## Graphical Abstract

**Graphical Abstract:**
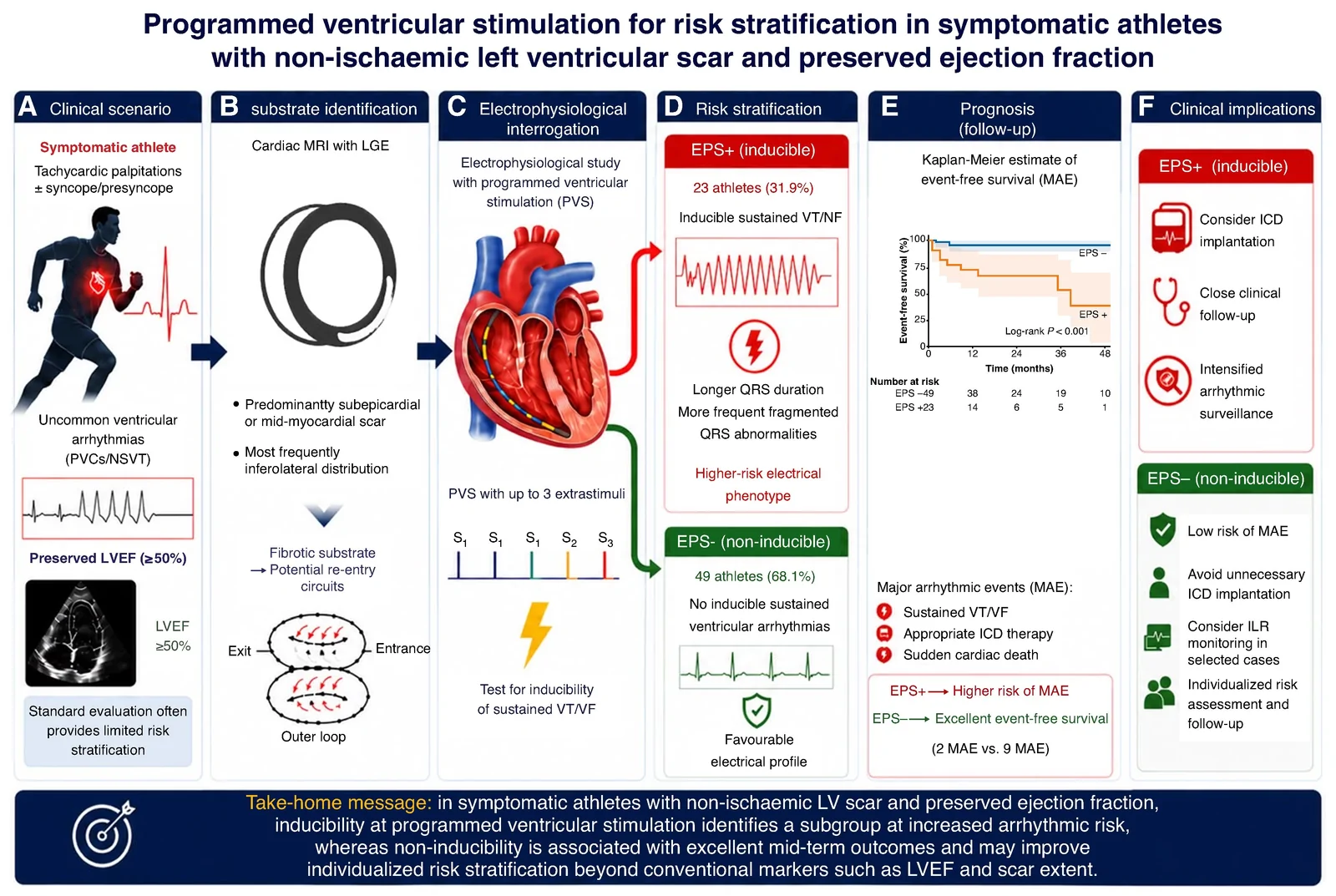

What’s New?

- This study provides new evidence on the prognostic value of programmed ventricular stimulation in symptomatic athletes with non-ischaemic left ventricular scar and preserved left ventricular ejection fraction.
- Inducibility at electrophysiological study was associated with a substantially higher rate of major arrhythmic events, whereas non-inducibility identified athletes with excellent mid-term event-free survival.
- Programmed ventricular stimulation may complement clinical and imaging findings to support individualized arrhythmic risk stratification in this population.
- The findings support the use of electrophysiological study as an adjunctive tool in the clinical evaluation of selected symptomatic athletes with non-ischaemic left ventricular scar.

## Background

Non-ischaemic left ventricular (LV) myocardial scar is increasingly recognized in athletes, even in the absence of overt structural heart disease. The widespread use of cardiac magnetic resonance imaging with late gadolinium enhancement has enabled the detection of focal myocardial fibrosis in athletes presenting with palpitations or apparently idiopathic ventricular arrhythmias,^1–3^ raising important questions regarding arrhythmic risk stratification, sports eligibility and the optimal diagnostic work-up of athletes with myocardial scar.^4*–*6^

Palpitations are among the most common reasons for cardiologic evaluation in athletes and may reflect premature ventricular complexes or non-sustained ventricular tachycardia.^7^ Although these arrhythmias are often considered benign in structurally normal hearts, the presence of a non-ischaemic LV scar represents a potential substrate for re-entrant ventricular tachyarrhythmia, with possible prognostic implications, including an increased risk of sudden cardiac death during intense physical activity. However, the role of electrophysiological study (EPS) with programmed ventricular stimulation (PVS) in arrhythmic risk stratification within this specific clinical setting remains poorly defined.^8^ The concept of invasive arrhythmic risk stratification through programmed ventricular stimulation has a long history. Initial studies performed in patients with previous myocardial infarction demonstrated the ability of programmed electrical stimulation to identify individuals at increased risk of sustained ventricular arrhythmias and sudden cardiac death, ultimately leading to the development of landmark risk stratification strategies and trials. In the athletic population, Heidbuchel *et al*.^9^ first highlighted the potential role of electrophysiological study as a discriminator of arrhythmic risk in athletes with structural heart disease. However, data regarding the prognostic value of inducibility in athletes with non-ischaemic left ventricular scar and preserved left ventricular function remain scarce.

The present study aimed to evaluate the role of PVS in arrhythmic risk stratification in a consecutive cohort of athletes with documented non-ischaemic LV scar and palpitations who underwent invasive EPS. Specifically, we assessed the prevalence of inducible sustained ventricular arrhythmias, the clinical and imaging correlates of inducibility, and its potential prognostic significance during follow-up.

By focusing on this highly relevant sports cardiology population, our study seeks to clarify the clinical value of ventricular arrhythmia inducibility and to inform risk stratification, therapeutic decision-making, and recommendations regarding continued participation in sports.

## Materials and methods

### Study population

This was a prospective, observational study including consecutive competitive and non-competitive athletes affected by arrhythmic symptoms and documented uncommon ventricular arrhythmias, with evidence of a non-ischaemic LV scar, referred to our institution for cardiologic evaluation, according to Italian Guidelines.^10–12^ All athletes were actively engaged in regular sports activity at the time of enrolment, with at least 5 years of sports participation and a minimum training load of 6 h per week. Only two athletes were professional competitors (one cyclist and one padel player). According to sport discipline classification,^11^ 35 athletes participated in endurance sports, 25 in mixed sports, and 12 in skill sports.

#### Eligibility criteria included:

- Presence of tachycardic palpitations with or without syncope/presyncope;
- Evidence of a non-ischaemic LV myocardial scar detected by cardiac magnetic resonance (CMR) with late gadolinium enhancement (LGE). A non-ischaemic LV scar pattern was defined as LGE with a non-coronary distribution (i.e. subepicardial or mid-myocardial), not consistent with ischaemic heart disease.

Asymptomatic athletes with incidentally detected non-ischaemic LV scar were not routinely referred for invasive electrophysiological evaluation during the study period, as current sports cardiology recommendations and contemporary ventricular arrhythmia guidelines do not support the routine use of EPS in this clinical setting.

#### Exclusion criteria

Athletes with a history of prior myocardial infarction, obstructive coronary artery disease, significant valvular heart disease, or overt cardiomyopathy with reduced left ventricular ejection fraction (LVEF <50%) were excluded.

The exclusion of ischaemic heart disease was based on clinical assessment and CMR tissue characterization; coronary CT angiography was performed in selected cases when clinically indicated to exclude coronary artery disease or coronary anomalies.

All participants provided written informed consent. The study was conducted in accordance with the Declaration of Helsinki and was approved by the Internal Ethical Board of our Institution.

### Clinical and non-invasive evaluation

All athletes underwent a comprehensive clinical and instrumental assessment including:

Resting 12-lead electrocardiogram (ECG)

Transthoracic echocardiography with colour Doppler

24-hour ambulatory ECG (Holter monitoring)

Genetic testing

Maximal symptom-limited exercise testing

Electrocardiographic abnormalities were defined according to current recommendations.^13^ Fragmented QRS (fQRS) was defined as the presence of additional R waves (R′) or notching in the nadir of the S wave in at least two contiguous leads.^14^ T-wave inversion (TWI) was defined as negative T waves ≥1 mm in at least two contiguous leads. Low QRS voltages were defined according to standard criteria.^15^

Genetic testing was performed using next-generation sequencing multigene panels for inherited cardiomyopathies and arrhythmia syndromes. The extended panel included more than 200 genes associated with cardiomyopathies, channelopathies, and inherited arrhythmic disorders. The complete list of genes included in the panel is provided in Supplementary Appendix 1. Variants were classified according to current international recommendations.^16^

Exercise testing was performed using a standardized cycle ergometer protocol. Maximal effort was defined as achievement of >90% of the age-predicted maximal heart rate. Ventricular arrhythmias during exercise and recovery were systematically recorded. Premature ventricular complexes (PVCs), non-sustained ventricular tachycardia (NSVT), arrhythmic burden, and morphology were analysed according to current recommendations.^17^

### CMR findings

CMR studies, evaluated for the enrolment, were performed using a 1.5-T scanner. Standard cine sequences were acquired to assess LV volumes, mass, and systolic function. Late gadolinium enhancement imaging was obtained 10–15 min after intravenous administration of gadolinium-based contrast (0.1–0.2 m mol/kg). Scar localization (apical, septal, lateral, inferior, anterior, ring-like), pattern (subepicardial, mid-wall, or transmural), and extension were systematically evaluated. For segmental analysis, the left ventricle was divided according to the standardized 17-segment American Heart Association model.^18^ The presence, localization, and pattern of late gadolinium enhancement were assessed for each segment. Scar extent was semi-quantitatively evaluated by visually estimating the percentage of segmental wall thickness exhibiting late gadolinium enhancement and categorized as ≤50%, or >50% of myocardial wall thickness. In addition, scar extent was assessed semi-quantitatively as the number of left ventricular segments showing late gadolinium enhancement according to the 17-segment American Heart Association model. LVEF was calculated using Simpson’s method from short-axis cine images.

### Electrophysiological study and programmed ventricular stimulation

All athletes underwent an invasive electrophysiological study (EPS). Programmed ventricular stimulation was performed from the right ventricular apex and right ventricular outflow tract using up to three extrastimuli delivered at a minimum drive cycle length of 400 ms. Coupling intervals were progressively shortened until ventricular refractoriness was reached, but never below 200 ms. No catecholamine infusion was administered during the study in order to avoid non-specific pharmacologically facilitated inducibility. The stimulation protocol included both right ventricular sites; however, in athletes who became inducible during stimulation from the first site, completion of the protocol from the second site was not considered necessary. Inducibility was defined as the induction of sustained monomorphic or polymorphic ventricular tachycardia (lasting ≥30 s or requiring termination due to haemodynamic instability) or ventricular flutter or ventricular fibrillation. The morphology and cycle length of induced arrhythmias were recorded.

### Follow-up and study endpoints

Following EPS, all inducible athletes underwent ICD implantation (transvenous or subcutaneous) as part of the clinical management strategy. Non-inducible athletes underwent clinical follow-up with or without implantable loop recorder monitoring according to shared clinical decision-making. All athletes were advised to discontinue competitive sports activity after evaluation and to perform only low- to moderate-intensity recreational exercise during follow-up, in accordance with contemporary sports cardiology recommendations and individualized clinical assessment. ICD programming was individualized according to contemporary recommendations.^19^ Detection zones were set above the maximal heart rate achieved during exercise testing, which was routinely performed to optimize device programming and avoid inappropriate therapies. The primary outcome was the occurrence of a major arrhythmic event (MAE), defined as either of the following: sudden death, documented sustained VT or VF, or appropriate ICD therapy. SCD was defined, according to the World Health Organization definition, as unexpected death, not attributable to an extracardiac cause, within 1 h of the onset of new or worsening symptoms (witnessed arrest) or, if unwitnessed, within 24 h of last being seen in good health.^20^

### Statistical analysis

Continuous variables are expressed as mean ± standard deviation or median (interquartile range), as appropriate. Categorical variables are presented as counts and percentages. Comparisons between groups (inducible vs. non-inducible athletes) were performed using Student’s *t*-test or the Mann–Whitney *U* test for continuous variables and the chi-square or Fisher’s exact test for categorical variables. A two-sided *P*-value <0.05 was considered statistically significant.

Time-to-event analysis was performed using the Kaplan–Meier method. The primary endpoint was event-free survival, defined as time from baseline evaluation to first occurrence of the event of interest. Athletes without events were censored at the time of the last follow-up. The analysis was truncated at 50 months due to the limited number of athletes with longer follow-up. Differences between groups were assessed using the log-rank test. Results are reported as two-sided *P*-values, with *P* < 0.05 considered statistically significant. Analyses were performed using R software (survival and survminer packages).

## Results

A total of 72 athletes (32 competitive and 40 non-competitive) fulfilled eligibility criteria and were included in the study. They underwent an electrophysiological study (EPS): 23 (31.9%) were inducible (EPS+) and 49 (68.1%) were non-inducible (EPS−). At baseline, all inducible athletes underwent ICD implantation following a positive EPS (18 transvenous and 5 subcutaneous devices). Among non-inducible athletes, 16 received an implantable loop recorder for longitudinal rhythm monitoring, whereas the remaining athletes underwent clinical follow-up alone according to shared clinical decision-making. During follow-up, two non-inducible athletes underwent ICD implantation after experiencing a resuscitated major arrhythmic event, while five additional non-inducible athletes subsequently received an ICD because of recurrent non-sustained ventricular tachycardia associated with perceived arrhythmic risk.

### Genetic testing

Genetic testing was proposed to all 72 athletes enrolled in the study. Of these, 12 athletes declined testing. Among the 60 athletes who underwent genetic analysis, 28 (46.7%) showed no relevant genetic variants. Pathogenic or likely pathogenic variants were identified in 14 athletes (23.3%). The most frequently involved genes were DSP (4/60, 6.7%), LMNA (3/60, 5.0%), and RBM20 (3/60, 5.0%), followed by TNNT2 (2/60, 3.3%), DSG2 (1/60, 1.7%), and MYBPC3 (1/60, 1.7%).

In addition, variants of uncertain significance (VUS) potentially associated with cardiomyopathic phenotypes were detected in 12 athletes (20.0%), involving mainly DTNA (6/60, 10.0%), LDB3 (3/60, 5.0%), and MYL genes (3/60, 5.0%). Six further athletes (10.0%) carried benign or likely benign variants.

Pathogenic or likely pathogenic variants were more frequently observed among inducible athletes. EPS inducibility was documented in 9 of 14 athletes (64.3%) carrying pathogenic or likely pathogenic variants, compared with 14 of the remaining 58 athletes without identified pathogenic or likely pathogenic variants or without available genetic testing (24.1%).

### Baseline characteristics

Baseline clinical characteristics were largely comparable between groups (*Table 1*). Mean age at diagnosis was 48.9 ± 13.1 years, with a predominance of male sex (66.7%). A higher proportion of males was observed in the EPS + group compared with EPS− (82.6% vs. 59.2%), although this did not reach statistical significance (*P* = 0.066). No significant differences were found in anthropometric parameters, cardiovascular risk factors, or pharmacological therapy. Most athletes received beta-blocker therapy, titrated to the maximally tolerated dose according to clinical judgement and individual tolerance.

**Table 1:** Baseline characteristics of athletes undergoing electrophysiological study

Variable	All athletes (*n* = 72)	EPS + (*n* = 23)	EPS− (*n* = 49)	*P* value
Age at diagnosis, years	48.9 ± 13.1	47.3 ± 12.9	49.7 ± 13.8	0.57
Male sex	48 (66.7%)	19 (79.2%)	29 (59.2%)	0.07
Height, cm	172.9 ± 12.9	176.1 ± 7.6	171.4 ± 14.6	0.08
Weight, kg	77.2 ± 16.8	79.1 ± 12.4	76.3 ± 18.6	0.46
Body surface area, m^2^	1.91 ± 0.17	1.95 ± 0.16	1.89 ± 0.18	0.20
LVEF, %	57.5 ± 7.4	55.5 ± 5.4	58.4 ± 8.0	0.09
Competitive athletes, *n* (%)	32 (44.4%)	14 (60.9%	18 (36.7%)	0.08
**Cardiac comorbidities**				
Hypertension	23 (31.9%)	7 (30.4%)	16 (32.7%)	0.84
Smoking history	18 (25.0%)	4 (17.4%)	14 (28.6%)	0.38
Dyslipidaemia	29 (40.3%)	11 (47.8%)	18 (36.7%)	0.36
Diabetes mellitus	4 (5.6%)	2 (8.7%)	2 (4.1%)	0.59
**Pharmacological therapy**				
Beta-blocker	63 (87.5%)	20 (87.0%)	43 (87.8%)	0.92
ACE inhibitor or ARB	26 (36.1%)	8 (34.8%)	18 (36.7%)	0.87
**Indication for EPS**				
Tachycardic palpitations	38 (52.8%)	11 (47.8%)	27 (55.1%)	
Presyncope/syncope	28 (38.9%)	9 (39.1%)	19 (38.8%)	
Both	6 (8.3%)	3 (13.0%)	3 (6.1%)	
Overall comparison				0.62
**CMR LGE distribution**				
Septal LGE	26 (36.1%)	9 (37.5%)	17 (35.4%)	1.00
Anterior LGE	7 (9.7%)	2 (8.3%)	5 (10.4%)	1.00
Inferior LGE	38 (52.8%)	16 (66.7%)	22 (45.8%)	0.13
Lateral LGE	38 (52.8%)	12 (50.0%)	26 (54.2%)	0.80
Ring-like LGE	1 (1.4%)	1 (4.2%)	0 (0.0%)	0.33
Number of LGE-positive LV segments, median (IQR)	2 (1–3)	3 (2–3.75)	2 (1–2.25)	0.01

Clinical, demographic, and imaging characteristics of the overall cohort and stratified by inducibility (EPS + vs. EPS−). Continuous variables are expressed as mean ± SD and categorical variables as *n* (%).

Abbreviations: LVEF, left ventricular ejection fraction; LGE, late gadolinium enhancement.

Data are presented as **mean ± SD** or ***n* (%)**.

Left ventricular ejection fraction was preserved in both groups, with a non-significant trend towards lower values in the inducible group (55.5 ± 5.4% vs. 58.4 ± 8.0%, *P* = 0.092).

### Cardiac MRI findings

Late gadolinium enhancement (LGE) distribution was similar between groups. Inferior LGE was more frequently observed in EPS + group (66.7% vs. 45.8%), although this did not reach statistical significance (*P* = 0.134). No differences were found for septal, anterior, lateral, or multi-segment LGE involvement. CMR did not reveal evidence of overt right ventricular involvement, as no athlete exhibited right ventricular late gadolinium enhancement or regional wall motion abnormalities. Scar extent was additionally assessed according to the number of LGE-positive left ventricular segments. Inducible athletes exhibited a significantly greater extent of myocardial fibrosis compared with non-inducible athletes, with a median of 3 (IQR 2–3.75) vs. 2 (IQR 1–2.25) LGE-positive segments, respectively (*P* = 0.014).

### ECG parameters and abnormalities

Among ECG parameters, QRS duration was significantly longer in EPS + group (104.6 ± 15.2 ms vs. 95.5 ± 14.3 ms, *P* = 0.019), whereas PR interval and QTc interval did not differ between groups (table2).

ECG abnormalities were common, occurring in 84.7% of athletes (*Table 2*). Fragmented QRS (fQRS) was significantly more prevalent in inducible athletes compared with non-inducible athletes (43.5% vs. 16.3%, *P* = 0.029). No significant differences were observed for other ECG abnormalities, although T-wave inversion showed a non-significant trend towards higher prevalence in EPS + group (56.5% vs. 32.7%, *P* = 0.095).

**Table 2:** Electrocardiographic parameters and abnormalities in the study population

Variable	All athletes (*n* = 72)	EPS + (*n* = 23)	EPS− (*n* = 49)	*P* value
**ECG parameter**				
PR interval, ms	153.4 ± 32.2	157.0 ± 25.3	151.5 ± 35.4	0.45
QRS duration, ms	98.5 ± 15.1	104.6 ± 15.2	95.5 ± 14.3	0.02
QTc interval, ms	394.6 ± 14.0	394.3 ± 13.8	394.8 ± 14.3	0.90
**ECG abnormalities**				
└ Inferior fQRS	10 (13.9%)	6 (26.1%)	4 (8.2%)	0.09
└ Lateral fQRS	6 (8.3%)	3 (13.0%)	3 (6.1%)	0.59
└ Anterior fQRS	5 (6.9%)	3 (13.0%)	2 (4.1%)	0.37
Low QRS voltages	7 (9.7%)	1 (4.3%)	6 (12.2%)	0.53
T-wave inversion (any)	29 (40.3%)	13 (56.5%)	16 (32.7%)	0.10
└ Inferior TWI	18 (25.0%)	9 (39.1%)	9 (18.4%)	0.11
└ Lateral TWI	14 (19.4%)	7 (30.4%)	7 (14.3%)	0.20
└ Anterior TWI	9 (12.5%)	5 (21.7%)	4 (8.2%)	0.21
Pathological Q waves	6 (8.3%)	3 (13.0%)	3 (6.1%)	0.59
Poor R-wave progression	11 (15.3%)	6 (26.1%)	5 (10.2%)	0.16
Conduction disorders (any)	12 (16.7%)	5 (21.7%)	7 (14.3%)	0.50
└ RBBB	4 (5.6%)	1 (4.3%)	3 (6.1%)	1.00
└ LBBB	1 (1.4%)	0 (0%)	1 (2.0%)	1.00
└ LAFB	8 (11.1%)	3 (13.0%)	5 (10.2%)	0.70
└ LPFB	3 (4.2%)	2 (8.7%)	1 (2.0%)	0.24
└ RBBB + LAFB	2 (2.8%)	1 (4.3%)	1 (2.0%)	0.54
└ RBBB + LPFB	2 (2.8%)	0 (0%)	2 (4.1%)	1.00

Resting 12-lead ECG parameters and prevalence of electrocardiographic abnormalities in the overall cohort and according to inducibility at programmed ventricular stimulation (EPS + vs. EPS−). Continuous variables are expressed as mean ± standard deviation and categorical variables as *n* (%).

Abbreviations: ECG, electrocardiogram; fQRS, fragmented QRS; TWI, T-wave inversion; RBBB, right bundle branch block; LBBB, left bundle branch block; LAFB, left anterior fascicular block; LPFB, left posterior fascicular block.

### Maximal-exercise test

A non-sustained ventricular tachycardia (NSVT) was observed in 6.2% of the overall cohort. The prevalence of NSVT was slightly higher in inducible group compared to non-inducible one (9.1% vs. 5.8%); however, this difference was not statistically significant (*P* = 0.90).

### Indication for EPS

All athletes were symptomatic at presentation. The indication for EPS did not differ between groups (overall *P* = 0.62), with tachycardic palpitations being the most common presentation (52.8%), followed by presyncope/syncope (38.9%) and combined symptoms (8.3%). The clinical presentation leading to EPS referral was not significantly associated with inducibility. The distribution of tachycardic palpitations, presyncope/syncope, and combined symptoms was similar between inducible and non-inducible athletes (*Table 1*).

### Clinical outcomes

**Figure 1:**
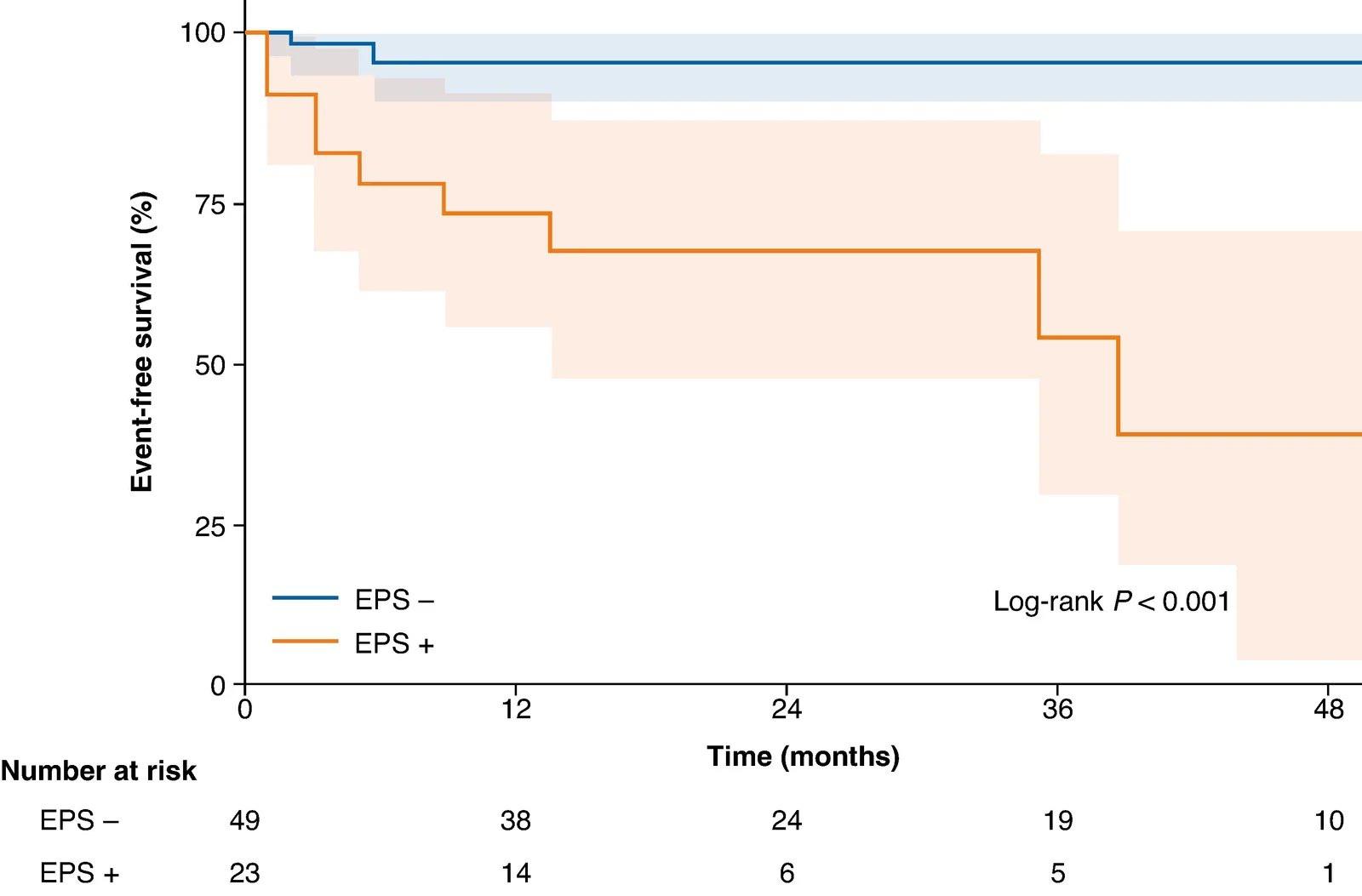
Kaplan–Meier estimates of event-free survival according to inducibility at electrophysiological study. Kaplan–Meier curves showing event-free survival according to inducibility at programmed ventricular stimulation during electrophysiological study (EPS + vs. EPS−) in symptomatic athletes with non-ischaemic left ventricular scar and preserved left ventricular ejection fraction. Survival analysis was based on time to first major arrhythmic event (MAE) only. Overall, 11 athletes experienced a first MAE during follow-up, including 9/23 inducible athletes and 2/49 non-inducible athletes. Downward steps indicate first MAE occurrence. Recurrent arrhythmic episodes and recurrent ICD therapies were not counted as additional events. Shaded bands represent 95% confidence intervals around Kaplan–Meier survival estimates. The analysis was truncated at 50 months because of the limited number of athletes remaining at risk beyond this timepoint. Numbers at risk are shown below the x-axis. Log-rank *P* < 0.001.

During a median follow-up of 42 months (minimum 8 months–maximum 148 months), 11 MAEs occurred: 9 among inducible athletes and 2 among non-inducible athletes. Kaplan–Meier analysis (*Figure 1*) demonstrated a significantly lower event-free survival among inducible athletes compared with non-inducible athletes (40.6% vs. 95.9% at 50 months; log-rank *P* < 0.001).

Of the nine MAEs occurring in inducible athletes (*Table 3*), all consisted of fast sustained ventricular arrhythmias detected within the programmed ventricular fibrillation zone and successfully terminated by appropriate ICD therapy, including ATP delivered during capacitor charging and shock therapy. Given their rapid rate and detection within the ventricular fibrillation zone, these events were considered potentially life-threatening ventricular arrhythmic events and were included in the composite study endpoint. Among non-inducible athletes, two MAEs required successful resuscitation and subsequently led to ICD implantation. No arrhythmic deaths occurred during follow-up. Six of the 11 major arrhythmic events (54.5%) occurred during moderate-intensity physical activity, whereas the remaining five events occurred at rest or were not exercise-related.

**Table 3:** Characteristics of athletes with major arrhythmic events

Sex	Age, years	Time to first event, months	LGE distribution		Genetic testing	Indication for EPS	EPS result	Induction protocol	Arrhythmia induced	ICD implantation	Major arrhythmic event
M	55	5	Infero-septal	Meso-subepicardial	TNNT	Palpitations	Inducible	S2	VFl	Yes	VF
M	59	1	Infero-lateral	Subepicardial	TNNT	Syncope	Inducible	S3	MSVT	Yes	MSVT
M	65	6	Infero-septal	Subepicardial	LMNA	Palpitations	Non inducible	S3	None	Yes	PSVT
F	64	14	Infero-lateral	Meso-subepicardial	No pathogenic variant detected	Palpitations and Syncope	Inducible	S2	MSVT	Yes	MSVT
M	61	2	Septal	Mesocardial	LMNA	Palpitations	Non inducible	S3	None	Yes	PSVT
F	44	10	Infero-lateral	Subepicardial	DSP	Palpitations	Inducible	S3	VF	Yes	VF
M	65	3	Infero-lateral	Meso-subepicardial	No pathogenic variant detected	Syncope	Inducible	S2	MSVT	Yes	MSVT
F	58	1	Septal; Apical	Meso-subepicardial	RBM20	Palpitations and Syncope	Inducible	S1	MSVT	Yes	MSVT
M	51	3	Infero-lateral	Meso-subepicardial	DSP	Palpitations and Syncope	Inducible	S2	PSVT	Yes	PSVT
M	50	40	Septal	Meso-subepicardial	No pathogenic variant detected	Palpitations	Inducible	S3	VFl	Yes	PSVT
M	22	36	Septal; Apical	Meso-subepicardial	No pathogenic variant detected	Palpitations	Inducible	S1	VF	Yes	VF

Clinical, imaging, electrophysiological, and genetic features of athletes who experienced major arrhythmic events during follow-up.

Abbreviations: VFl, ventricular flutter; MSVT, monomorphic sustained ventricular tachycardia; PSVT, polymorphic sustained ventricular tachycardia; VF, ventricular fibrillation

## Discussion

### Main finding

The main finding of the present study is that inducibility at EPS identifies a subgroup of symptomatic athletes with non-ischaemic LV scar and preserved ejection fraction at significantly increased risk of major arrhythmic events (MAE), whereas non-inducibility is associated with a very low event rate. These findings highlight the potential role of PVS as a clinically useful tool for arrhythmic risk stratification in this challenging and increasingly recognized population.

### Interpretation of results

In our cohort of symptomatic athletes, inducible ventricular arrhythmias were strongly associated with subsequent MAE during follow-up. Conversely, the low event rate observed among non-inducible athletes suggests a high negative predictive value of EPS in this setting. These findings are consistent with recent data by Soris *et al*.^21^ who demonstrated that inducibility is associated with increased arrhythmic risk in patients with myocardial scar and preserved or mildly reduced ejection fraction, with a negative predictive value approaching 97%.

Interestingly, inducibility and occurrence of MAE were more frequently observed among athletes carrying pathogenic or likely pathogenic genetic variants than among those without disease-causing variants. Although the study was not powered to evaluate genotype-specific risk, this finding suggests that EPS may help identify electrically vulnerable phenotypes within the spectrum of genetically mediated myocardial disease and supports the hypothesis that, in at least some athletes with non-ischaemic LV scar, myocardial fibrosis may represent the expression of an underlying concealed cardiomyopathic substrate. These findings are consistent with the contemporary concept that ventricular arrhythmias in athletes often reflect the interplay between myocardial fibrosis, genetic predisposition, and concealed structural heart disease, rather than representing a purely exercise-related phenomenon.^22^ Larger studies are warranted to further clarify the relationship between genotype, myocardial scar, and arrhythmic risk. Taken together, these observations support the concept that EPS provides incremental prognostic information beyond LVEF, which is known to be an imperfect marker of arrhythmic risk, particularly in individuals with preserved systolic function. Future advances in risk stratification may derive from integrating CMR-derived scar characterization with electroanatomic mapping, allowing a more comprehensive characterization of the arrhythmogenic substrate. Such an approach may also support catheter ablation planning in selected athletes with recurrent ventricular arrhythmias.^23^ Notably, more than half of the major arrhythmic events occurred during moderate-intensity physical activity. Although all athletes had discontinued competitive sports participation, this finding is consistent with previous observations suggesting that physical exertion may act as a trigger for malignant ventricular arrhythmias in susceptible individuals with an underlying arrhythmogenic substrate, including myocardial fibrosis.^24^ In athletes, in whom physiological remodelling may further complicate risk stratification, EPS may help identify both high-risk individuals who may benefit from closer monitoring or device therapy and low-risk athletes in whom unnecessary interventions and overly restrictive management strategies may be avoided.

### Relationship with previous studies

The use of programmed ventricular stimulation for arrhythmic risk stratification originated from studies performed in patients with previous myocardial infarction during the late 1970s and early 1980s, demonstrating that inducible ventricular arrhythmias identify individuals at increased risk of subsequent malignant ventricular events.^25,26^ These observations ultimately contributed to the development of landmark studies such as MUSTT^27^ and MADIT,^28^ establishing the role of invasive electrophysiological assessment in selected high-risk populations. In athletes, Heidbuchel *et al*.^9^ were among the first to demonstrate the potential value of electrophysiological study for risk stratification in individuals with structural heart disease, reporting outcome differences according to inducibility status.

Beyond the specific population investigated in the present study, contemporary evidence indicates that the substrates underlying sports-related sudden cardiac death are highly heterogeneous and frequently include concealed myocardial disease that may remain undetected despite conventional pre-participation evaluation.^29–32^ Although inherited cardiomyopathies and channelopathies remain important causes of sudden cardiac death in young athletes, increasing evidence supports a relevant role for non-ischaemic myocardial fibrosis as an arrhythmogenic substrate capable of triggering malignant ventricular arrhythmias during exercise. These observations highlight the limitations of traditional risk assessment strategies based primarily on clinical history, electrocardiography, and ventricular function, particularly in athletes with preserved systolic function and subtle structural abnormalities.

Data regarding invasive risk stratification in patients with preserved LVEF remain limited. The PRESERVE-EF study^33^ extended the role of EPS to post-myocardial infarction patients with LVEF ≥40%, while Soris *et al*.^21^ subsequently demonstrated the prognostic value of PVS in patients with myocardial scar and preserved ventricular function. Our study extends these observations to a more specific and clinically relevant population of symptomatic athletes with non-ischaemic LV scar, supporting the concept that inducibility identifies a subgroup with substantially higher arrhythmic risk despite preserved LVEF.

### Role of myocardial scar and electrical substrate

Myocardial scar represents the key arrhythmogenic substrate in athletes with ventricular arrhythmias and preserved LVEF and likely explains the limited performance of LVEF-based risk stratification in this population. In the present study, inducible athletes exhibited a greater extent of myocardial fibrosis, as reflected by a higher number of LGE-positive left ventricular segments compared with non-inducible athletes. This finding is consistent with previous observations linking scar burden to ventricular arrhythmogenicity and adverse outcomes.^34,35^

However, scar extent alone is unlikely to fully explain the marked prognostic separation observed between inducible and non-inducible athletes. Indeed, electrocardiographic markers of electrical instability, particularly fragmented QRS, were also more prevalent among inducible athletes, suggesting that both the anatomical substrate and its electrophysiological properties contribute to arrhythmic vulnerability. These findings support the concept that EPS provides complementary information beyond structural characterization alone, identifying athletes in whom myocardial scar is associated with a clinically relevant arrhythmogenic substrate.

An additional consideration is the heterogeneous nature of non-ischaemic myocardial fibrosis. Although all athletes included in the present study shared the presence of a non-ischaemic left ventricular scar, the underlying substrates likely encompassed different aetiologies, including post-inflammatory fibrosis, genetically mediated cardiomyopathies, and idiopathic scar patterns. These conditions may differ in terms of arrhythmogenic potential, electrophysiological properties, and clinical outcomes. Owing to the limited sample size, the present study was not designed to evaluate inducibility according to scar aetiology. Future investigations involving larger cohorts and more detailed substrate characterization will be required to determine whether the prognostic significance of inducibility varies across different scar phenotypes.

### Clinical implications

The present findings have important implications for clinical practice, particularly in the evaluation of athletes with ventricular arrhythmias and evidence of myocardial scar.

The present findings are also consistent with the paradigm proposed by the recent Lancet Commission on Sudden Cardiac Death,^36^ which advocates a transition from traditional population-based approaches towards precision prevention strategies integrating structural, electrical, genetic, and clinical markers of risk. In this context, inducibility at EPS may represent an additional component of individualized arrhythmic risk assessment in athletes with preserved LVEF and myocardial fibrosis.

Current guidelines for primary prevention of sudden cardiac death rely largely on LVEF thresholds, despite evidence that a substantial proportion of events occur in individuals with preserved or mildly reduced systolic function.^37^

In athletes, risk stratification is further complicated by the overlap between physiological cardiac remodelling and early cardiomyopathic changes. Within this challenging clinical scenario, our results confirm that EPS may represent a valuable adjunctive tool for refining risk stratification in symptomatic athletes with non-ischaemic LV scar.

In particular, the high negative predictive value observed in non-inducible athletes may help identify individuals at low risk, potentially avoiding unnecessary implantable cardioverter-defibrillator (ICD) implantation and unwarranted restriction from competitive sports. In these athletes, implantable loop recorder monitoring may represent a valuable strategy for longitudinal arrhythmic surveillance and shared decision-making when ICD implantation is not considered justified.

If confirmed in larger prospective studies, the excellent event-free survival observed among non-inducible athletes may contribute to further refining risk assessment strategies and eligibility criteria for sports participation in athletes with non-ischaemic LV scar. Conversely, inducible athletes represent a higher-risk subgroup in whom intensified surveillance, lifestyle modification, and consideration of device therapy may be warranted.

It should be emphasized that all athletes included in the present study were symptomatic and underwent electrophysiological evaluation because of a clinical indication. At present, neither sports cardiology recommendations nor contemporary ventricular arrhythmia and cardiomyopathy guidelines support the routine use of programmed ventricular stimulation in asymptomatic athletes with incidentally detected non-ischaemic myocardial scar. Although the marked prognostic separation observed in our cohort raises the hypothesis that EPS may also have a role in selected asymptomatic individuals, this question remains unanswered and should be addressed in dedicated prospective studies specifically designed for that population.

Importantly, EPS should not be used in isolation but rather integrated with clinical presentation, imaging findings, genetic information, and electrocardiographic markers, consistent with contemporary multiparametric approaches to arrhythmic risk stratification.^6,32^

## Conclusions

In symptomatic athletes with non-ischaemic LV scar and preserved ejection fraction, inducibility of sustained ventricular arrhythmias during EPS is associated with a higher risk of major arrhythmic events during follow-up, whereas non-inducibility identifies a subgroup with a low event rate.

These findings suggest that PVS may provide incremental information for arrhythmic risk stratification beyond conventional markers such as LVEF, particularly in this selected population.

However, given the observational design and the relatively small sample size, these results should be considered hypothesis-generating and warrant confirmation in larger prospective studies.

## Limitations

This study has several limitations. First, the observational design may introduce selection bias, as only symptomatic patients referred for electrophysiological evaluation were included. Second, the study population consisted mainly of middle-aged athletes, which may limit the generalizability of the findings to other clinical settings. Third, although the composite endpoint reflects clinically relevant outcomes, its heterogeneous nature should be considered when interpreting the results. Fourth, quantitative scar burden expressed as a percentage of left ventricular mass was not systematically available and therefore could not be included in the present analysis. Scar extent was instead assessed using a semi-quantitative segmental approach based on the 17-segment AHA model. Future prospective studies incorporating standardized quantitative scar assessment may further clarify the relationship between myocardial fibrosis burden, inducibility, and clinical outcomes. Additionally, because ICD implantation was guided by clinical risk assessment and EPS findings, inclusion of appropriate ICD therapies in the composite endpoint may have increased event ascertainment among ICD recipients, although most device interventions were delivered for fast ventricular arrhythmias detected within the ventricular fibrillation zone. Finally, the limited number of events precluded reliable multivariable Cox regression analyses.

## Supplementary Material

euag160_Supplementary_Data

## Data Availability

The data underlying this article will be shared on reasonable request to the corresponding author. The data are not publicly available because they contain information that could compromise the privacy of research participants.
