# Supplementary material for: Programmed ventricular stimulation for risk stratification in symptomatic athletes with nonischemic left ventricular scar and preserved ejection fraction
Source: Europace. 2026 Sep 8;28(9):euag160. doi: 10.1093/europace/euag160 (PMC13550935; doi:10.1093/europace/euag160)
Supplement: Supplementary file 1 [file euag160_supplementary_data.docx]

**Supplementary Table S1 Baseline characteristics according to occurrence of major arrhythmic events (MAE) during follow-up.**

Data are presented as mean values, median values, or number (%) as appropriate. Genetic percentages were calculated among athletes who underwent genetic testing.

| **Variable** | **MAE (n=11)** | **No MAE (n=61)** |
| --- | --- | --- |
| Age at diagnosis, years | 54.0 ± 12.6 | 48.0 ± 13.1 |
| Male sex, n (%) | 8 (72.7%) | 40 (65.6%) |
| Beta-blocker, n (%) | 10 (90.9%) | 53 (86.9%) |
| Tachycardic palpitations, n (%) | 6 (54.5%) | 32 (52.5%) |
| Presyncope/syncope, n (%) (tot 28) | 2 (18.2%) | 26 (42.6%) |
| Both symphtoms, n (%) (tot 6) | 3 (27.3%) | 3 (4.9%) |
| LVEF, % | 56.5± 6.4 | 58.3± 6.9 |
| Competitive athletes, n (%) | 4 (36.4%) | 28 (45.9%) |
| EPS inducibility, n (%) | 9 (81.8%) | 14 (23.0%) |
| Pathogenic/likely pathogenic variant*, n/N tested (%) | 7/11 (63.6%) | 8/49 (16.3%) |
| Scar burden (median number of LGE-positive LV segments) | 3 | 2 |
| ICD implantation, n % | 11 (100%) | 19 (31.1%) |
|  |  |  |

* Percentages calculated among athletes who underwent genetic testing (11/11 in the MAE group and 49/61 in the non-MAE group).

**Supplementary Table S2: Comparison of demographic, athletic, imaging, electrophysiological, and clinical characteristics between competitive and non-competitive athletes.**

Continuous variables are presented as mean ± standard deviation (SD) or median (interquartile range [IQR]), as appropriate. Categorical variables are presented as number (percentage). Comparisons between groups were performed using the Student’s t-test or Mann–Whitney U test for continuous variables and the χ² test or Fisher’s exact test for categorical variables, as appropriate.

LVEF = left ventricular ejection fraction; LGE = late gadolinium enhancement; EPS = electrophysiological study; MAE = major arrhythmic event.

| **Variable** | **Competitive (n=32)** | **Non-competitive (n=40)** | **p** |
| --- | --- | --- | --- |
| Age, years | 47.0 ± 13.1 | 53.7 ± 11.5 | 0.03 |
| Weekly training load (h/week) | 9.5 ± 1.5 | 7.5 ± 1.5 | <0.001 |
| Endurance sport, n (%) | 15 (46.9) | 20 (50.0) |  |
| Mixed sport, n (%) | 12 (37.5) | 13 (32.5) |  |
| Skill sport, n (%) | 5 (15.6) | 7 (17.5) | 0.91* |
| EPS inducibility, n (%) | 14 (43.8) | 9 (22.5) | 0.076 |
| Major arrhythmic events, n (%) | 4 (12.5) | 7 (17.5) | 0.74 |
| LVEF, % | 57.0 ± 6.9 | 58.8 ± 7.5 | 0.30 |
| LGE-positive segments, median (IQR) | 2 | 2 | 1.00 |

* Global comparison among sport disciplines.

**Supplementary Appendix 1**

Complete list of genes included in the extended next-generation sequencing panel (>200 genes) used for the genetic evaluation of athletes enrolled in the study. The panel included genes associated with inherited cardiomyopathies, channelopathies, and arrhythmia syndromes.

A2ML1, ABCC9, ABCG5, ABCG8, ACTA1, ACTA2, ACTC1, ACTN2, AKAP9, ALMS1, ALPK3, ANK2, ANKRD1, APOA4, APOA5, APOB, APOC2, APOE, BAG3, BMPR2, BRAF, CACNA1C, CACNA2D1, CACNB2, CALM1, CALM2, CALM3, CALR3, CASQ2, CAV3, CAVIN4, CBL, CBS, CDH2, CETP, COL3A1, COL5A1, COL5A2, COX15, CREB3L3, CRELD1, CRYAB, CSRP3, CTF1, CTNNA3, DES, DMD, DNAJC19, DOLK, DPP6, DSC2, DSG2, DSP, DTNA, EFEMP2, ELN, EMD, EYA4, FBN1, FBN2, FHL1, FHL2, FKRP, FKTN, FLNA, FLNC, FXN, GAA, GATA6, GATAD1, GCKR, GJA5, GLA, GPD1L, GPIHBP1, HADHA, HCN2, HCN4, HFE, HRAS, HSPB8, ILK, JAG1, JPH2, JUP, KBTBD13, KCNA5, KCND3, KCNE1, KCNE2, KCNE3, KCNE5, KCNH2, KCNJ2, KCNJ5, KCNJ8, KCNQ1, KLF10, KRAS, KRT17, LAMA2, LAMA4, LAMP2, LDB3, LDLR, LDLRAP1, LGALS3, LMF1, LMNA, LOX, LPL, LTBP2, LZTR1, MAP2K1, MAP2K2, MIB1, MYBPC3, MYH11, MYH6, MYH7, MYL2, MYL3, MYLK, MYLK2, MYO6, MYOZ2, MYPN, NEXN, NKX2-5, NODAL, NOTCH1, NPPA, NRAS, PCSK9, PDLIM3, PKP2, PLN, PMEPA1, PPP1CB, PRDM16, PRKAG2, PRKAR1A, PTPN11, RAF1, RANGRF, RBM20, RIT1, RRAD, RYR1, RYR2, SALL4, SCN10A, SCN1B, SCN2B, SCN3B, SCN4B, SCN5A, SCO2, SDHA, SELENON, SGCB, SGCD, SGCG, SHOC2, SLC25A4, SLC2A10, SLMAP, SMAD3, SMAD4, SNTA1, SOS1, SOS2, SREBF2, TAZ, TBX20, TBX3, TBX5, TCAP, TECRL, TGFB2, TGFB3, TGFBR1, TGFBR2, TJP1, TMEM43, TMPO, TNNC1, TNNI3, TNNT2, TPM1, TRDN, TRIM63, TRPM4, TTN, TTR, TXNRD2, VCL, ZBTB17, ZHX3, ZIC3.
